# Sensitivity of Above-Ground Biomass Estimates to Height-Diameter Modelling in Mixed-Species West African Woodlands

**DOI:** 10.1371/journal.pone.0158198

**Published:** 2016-07-01

**Authors:** Rubén Valbuena, Janne Heiskanen, Ermias Aynekulu, Sari Pitkänen, Petteri Packalen

**Affiliations:** 1 School of Forest Sciences, University of Eastern Finland, Joensuu, Finland; 2 Department of Geosciences and Geography, University of Helsinki, Helsinki, Finland; 3 World Agroforestry Centre (ICRAF), Nairobi, Kenya; Kerala Forest Research Institute, INDIA

## Abstract

It has been suggested that above-ground biomass (*AGB*) inventories should include tree height (*H*), in addition to diameter (*D*). As *H* is a difficult variable to measure, *H*-*D* models are commonly used to predict *H*. We tested a number of approaches for *H*-*D* modelling, including additive terms which increased the complexity of the model, and observed how differences in tree-level predictions of *H* propagated to plot-level *AGB* estimations. We were especially interested in detecting whether the choice of method can lead to bias. The compared approaches listed in the order of increasing complexity were: (**B0**) *AGB* estimations from *D*-only; (**B1**) involving also *H* obtained from a fixed-effects *H*-*D* model; (**B2**) involving also species; (**B3**) including also between-plot variability as random effects; and (**B4**) involving multilevel nested random effects for grouping plots in clusters. In light of the results, the modelling approach affected the *AGB* estimation significantly in some cases, although differences were negligible for some of the alternatives. The most important differences were found between including *H* or not in the *AGB* estimation. We observed that *AGB* predictions without *H* information were very sensitive to the environmental stress parameter (*E*), which can induce a critical bias. Regarding the *H*-*D* modelling, the most relevant effect was found when species was included as an additive term. We presented a two-step methodology, which succeeded in identifying the species for which the general *H*-*D* relation was relevant to modify. Based on the results, our final choice was the single-level mixed-effects model (**B3)**, which accounts for the species but also for the plot random effects reflecting site-specific factors such as soil properties and degree of disturbance.

## Introduction

Forest is the main terrestrial biotic reservoir for above-ground carbon stock [1]. For this reason, accurate estimates of forest above-ground biomass (*AGB*) are required to understand the global carbon cycle, thereby to implement climate change mitigation policies [2], and support project activities such as REDD+ (Reducing Emissions from Deforestation and Forest Degradation in developing countries + conservation of forest carbon stocks, sustainable management of forest and enhancement of forest carbon stocks) [3]. Globally, substantial effort has been made to develop robust and cost-effective above-ground biomass estimation methods, including field measurements and remote sensing [4]. However, the accuracy in the estimation of *AGB* still lags behind the required level, and uncertainties in the terrestrial carbon storage are particularly large in the tropical areas [5]. In comparison to other regions of the world, the *AGB* estimates of the African forests and woodlands, however, remain scarce [6,7]. In terms of REDD+, large areas of savanna woodlands, shrublands and agroforestry parklands qualify as forests (e.g. [8]). Biomass typically constitutes the largest fraction of the primary energy consumption in these areas [7], and hence *AGB* estimates are important part of fuelwood resource assessments [9].

Although destructive sampling of above-ground biomass estimation is an accurate method (e.g., [9–17]), its implementation for carbon inventories is rarely practical and hence allometric models are commonly used instead (e.g., [1,4,6,18]). Since allometric models might be an important source of estimation uncertainty [19–22], the selection of the most suitable model from the literature requires special attention. Furthermore, using species-specific models is an unfeasible option in species-rich tropical countries [23] and therefore mixed species models for groups of similar species have to be used. In the pan-tropical models, species are often taken partly into account by including wood density (*ρ*) in the allometry [24], which can be either measured or extracted from the literature or online databases (e.g., [25]).

The most typical tree parameter used as predictor in *AGB* models is the diameter at breast height (*D*), because it is easy to measure with high accuracy in the field and, accordingly, it is usually available in the forest inventory databases. Other typically used parameters are total tree height (*H*), *ρ* and crown diameter. Allometric models based on *D* only are commonly used, but the importance of including tree height in biomass estimation has been emphasized by several authors [24,26–29]. The relationships between biomass and tree attributes depend on factors such as site, successional status, ecological zone, forest type and management, but *H*-*D* allometry can explain most of this variation [17]. Therefore, inclusion of *H* could improve model applicability to different sites [26]. Recently, Chave et al. [24] suggested that a single *AGB* model based on *D*, *H* and *ρ* holds across tropical vegetation types, regions and environmental factors.

Feldpausch et al. [27] showed that mean *AGB* of plots located across the tropics was 13% lower when including *H*, which implies that carbon storage estimate may be prone to bias if *H* is ignored. Also the mean error in tree-level biomass estimates was halved when accounting for *H*. Marshall et al. (2012) found that aboveground carbon was overestimated by 55 Mg ha^–1^ if *H* was not included in the allometric model in Tanzania, which would cause an overestimation of carbon resource by about 24%. Furthermore, simulations by Molto et al. [20] showed that a “2−5% bias in *H* predictions can propagate to *AGB* estimations off their own confidence intervals“. On the other hand, sometimes *AGB* model based on *D* only is recommended because such model is more parsimonious and applicable to inventory data that has only *D* measurements (e.g., [14]). Many authors have also found that inclusion of *H* to biomass model might only lead to negligible changes in biomass prediction [10,12,15,30,31]. Therefore, it is still an open question whether the inclusion of *H* in the allometry actually matters in terms of *AGB* estimation, under what circumstances or conditions, and what are the factors that can lead to relevant changes in the estimation.

Measuring *H* is difficult and expensive, especially in the tropics [13]. Therefore, to increase the cost-efficiency of the field work, it is a common procedure to measure *D* and identify species from every tree, but measure *H* just for a subset of trees, called sample trees [32]. The objective would then be to generalize the sample tree *H* to the rest of the trees, called tally trees. The *H*-*D* model is used for this purpose. There are numerous studies comparing the functional forms for the *H*-*D* relationship. According to Mehtätalo [33] the most frequently used function forms are the power function [34], Meyer’s equation [35] and the Korf curve [36]. A plethora of basic functional forms have been tested [37–39], but none of them has been found to be superior. The *H*-*D* relationships vary by tree species and therefore *H*-*D* curves should be fitted by species [40–42]. However, if the number of tree species is high, species-specific modeling is not a viable option. In that case, information accounting for species variation should be incorporated to the model itself [43,44].

Chave et al. [24] advised that for reliable *AGB* estimations it is required to develop local *H*-*D* allometry, by measuring total *H* on a subsample of the trees stratified by forest type and covering the range of diameters in full. The reason for this is grounded on the fact that the *H*-*D* relationship varies considerably between plots, stands and regions [37,45–47]. This variation in *H*-*D* curves is often taken into account by adjusting plot/stand/region-specific *H*-*D* curve parameters. There are, however, many alternatives to do the localization. A simple approach is to use either existing *H*-*D* model or to fit a local *H*-*D* model and then re-scale it so that the measured mean height at wanted level is obtained when the *D* equals to the measured mean diameter. Alternatively, more complex models can be adjusted by adding covariates known to modify the *H*-*D* relationship, such as site index [35], stem density [48], or other stand-level characteristics such as basal area or dominant height [49].

A more advanced statistical approach is to use a linear or non-linear mixed-effects modeling (NLME) [50]. NLME suits well for the generalization of sample tree information to tally trees because plot based inventory data is collected in a hierarchical manner (e.g. tree-plot-cluster) and random (e.g. plot-cluster) effects are available while predicting [45]. For this reason, mixed-effects modeling was suggested by Lappi and Bailey [51] as a method to predict stand development. The random effects allow taking into account the effect of multiple causal relations in the model [49,52] or the influence of unknown covariates [53] affecting the *H*-*D* relationship. They allow for developing models accounting for spatial variability in large-scale modelling [43,46,54]. For these reasons, mixed-effects modelling has become a widespread approach to *H*-*D* modelling [39,49,52,55,56].

In this study, our objective was to examine how sensitive *AGB* estimation in woodlands of the Sudanian savanna in Burkina Faso are to the use of allometry either omitting or including *H*, and the approach employed for *H*-*D* modelling. More specifically, first, we studied how accurately *H* can be predicted in a species-rich study site, using a small subset of trees measured for *H* and several options for non-linear fixed and mixed-effects models. Then, we examined the propagation of the *H* differences into the *AGB* predictions. With this research we wished to shed some light on whether the *H*-*D* modelling approach actually matters with regards to the target outcome: the *AGB* prediction. We also compared those *AGB* predictions with allometry not including *H* as predictor, and reflected on plausible alternatives to *H*-*D* modelling.

## Material and Methods

### Study area

**Fig 1:**
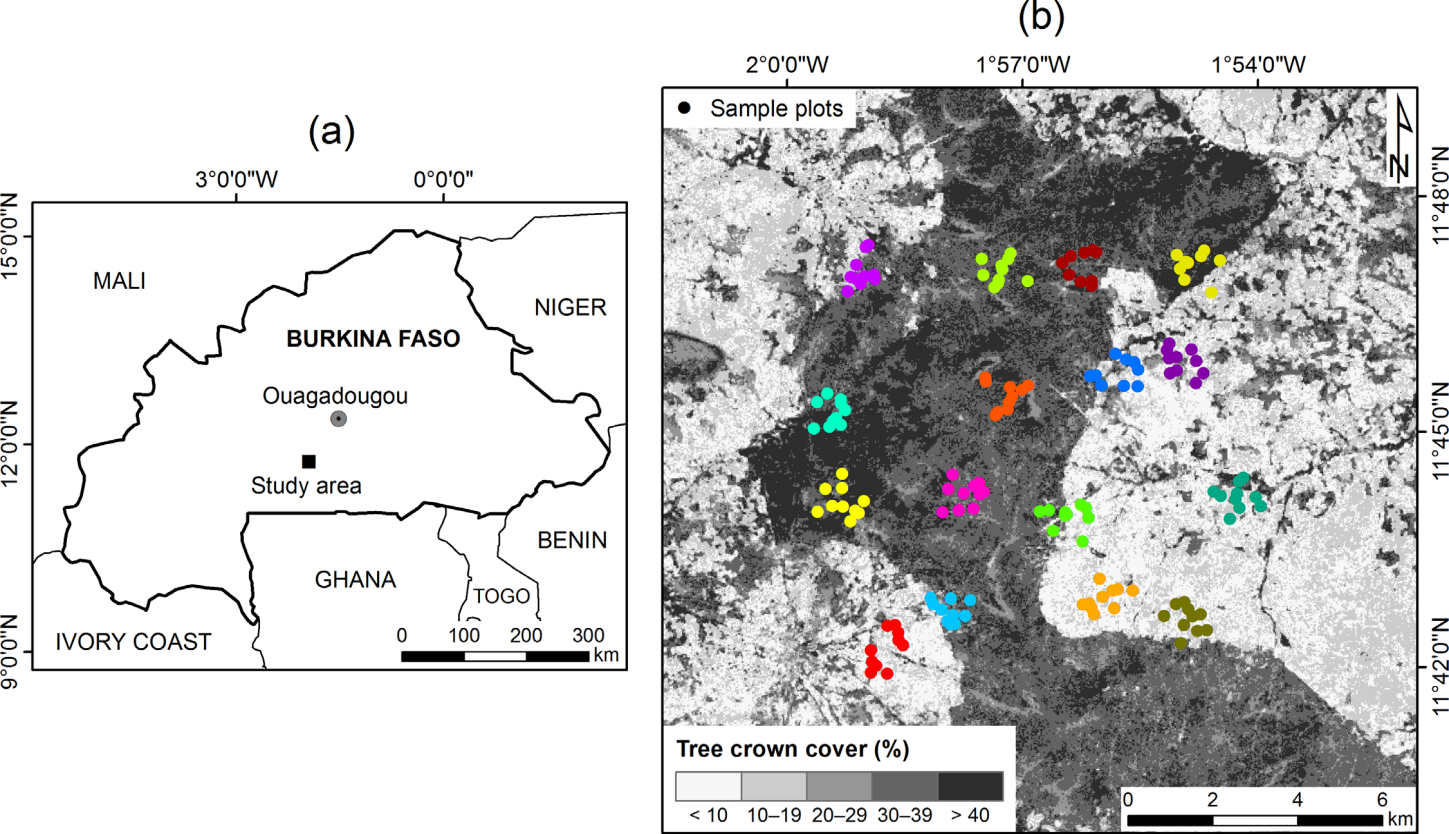
**(a) The location of the study area and (b) the distribution of the sample plots.** Different colours signify clusters in a sampling design which followed the Land Degradation Surveillance Framework (LDSF) [61,62]. The background reference is a map of crown cover predictions, courtesy of Jinxiu Liu (unpublished; see acknowledgments).

The study site is located in the southern Burkina Faso in the Ziro province (approx. 11°44'N 1°56'W) (Fig 1A). The area belongs to the West Sudanian savanna ecoregion [57]. According to the WorldClim dataset [58], the mean annual precipitation is 827 mm in 1950–2000 and the mean annual temperature is 27.5°C. The most of the precipitation falls between May and September, the wettest month being August. The driest months are December, January and February. Mean minimum and maximum temperatures were 24.7°C and 38.3°C in the warmest month (April) and 16.1°C and 33.6°C in the coldest month (December). In the Köppen-Geiger climate classification, the area lies in the transition of Arid steppe (BSh) and tropical savanna climate types (Aw) [59]. Topographically, the study area is relatively flat with a mean elevation of 350 m above sea level. Plinthosols with subsurface accumulation of ironoxides, kaolinitic clay and quartz (plinthite) are the dominant soil type [60].

The land cover in the study area consists of savanna woodlands with variable densities of tree crown cover, surrounded by settlements and agroforestry parklands, which are typically dominated by single species, like *Vitellaria paradoxa* [63]. Majority of the forest area is under community forest management and protection (Chantiers d'Aménagement Forestier, CAF), a 1986’s participatory program aiming at providing sustainable fuelwood production for the capital Ouagadougou which involves the local communities in the practical management of forests [64]. Furthermore, forests and woodlands are also partly used for livestock grazing [64]. Fires due to anthropogenic and natural causes take place regularly in the study area and influence the ecology of the vegetation (e.g., [65]).

### Field data collection

Field data consisted of 160 sample plots measured between December 2013 and February 2014 in the framework of Building Biocarbon and Rural Development in West Africa (BIODEV). Permissions for field work where given by Institut de l'Environnement et Recherches Agricoles (INERA). The sampling design was based on the Land Degradation Surveillance Framework (LDSF) [61,62]. The 10,000 ha (10 km × 10 km) site was stratified into 16 tiles, which were sampled by 100 ha clusters. Each cluster had ten 0.1 ha circular sample plots (radius 17.84 m). Cluster and sample plot centre points were randomly placed (Fig 1B). At each of 0.1 ha plots, diameters were measured at a height of 1.3 m above ground (*D*; cm) for the stems having *D* > 10 cm. Tree height (*H*; m) was measured for trees of smallest, median and largest diameter. Furthermore, all the stems having *D* = 4–10 cm were counted at four 0.01 subplots (radius 5.64 m) evenly located within each 0.1-ha plot [62], where also *D* and *H* were measured and species recorded for the median diameter tree. Diameters were measured using a diameter tape, and heights with a hypsometer (Suunto PM-5/1520, Vantaa, Finland). Tree species were determined for each stem by a field crew with local expertise.

**Fig 2:**
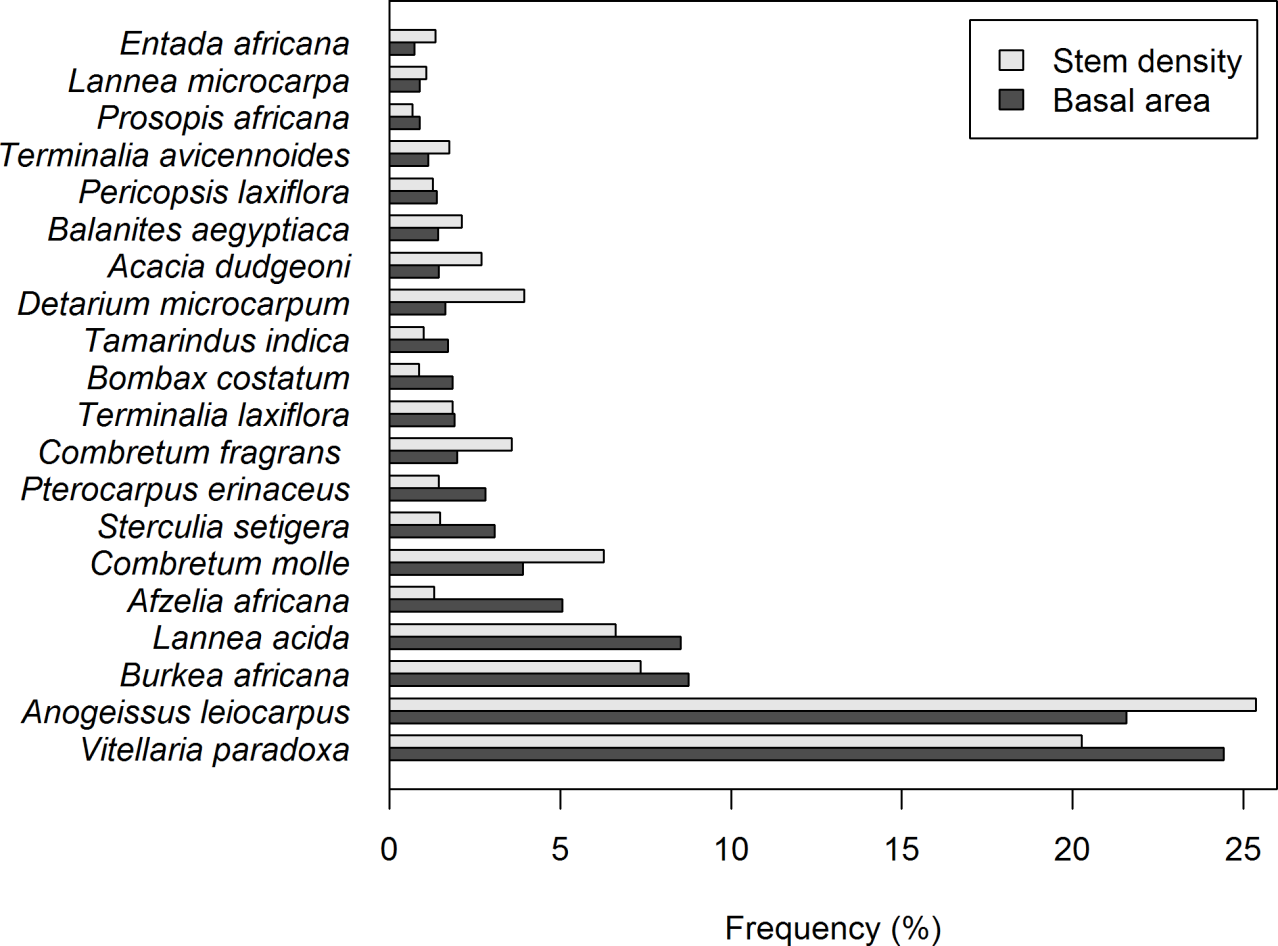
The frequencies of the twenty most common tree species ordered by the proportion of the total basal area. Frequencies of stems (proportions of total density) for those same species are also shown.

Out of those 160 plots, 152 plots had living trees. *D* was measured for a total of 2298 stems, and both *D* and *H* for 403 stems. Descriptive statistics for the sample plots are given in Table 1. A total of 54 tree species were observed, although the ten most common species accounted for 80% of the stems (Fig 2). *Anogeissus leiocarpus* and *Vitellaria paradoxa* were the most common tree species (Fig 2). In the trees sampled for *H* measurement, there were in total 36 tree species present.

**Table 1:** Descriptive statistics for the sample plots with trees (n = 152).

	Min	Max	Mean	SD
Stem density (stems ha^-1^)	10	1935	494	401
Mean diameter (cm)	6.4	40.0	15.2	8.5
Quadratic mean diameter (cm)	8.2	48.1	21.6	7.5
Basal area (m^2^ ha^-1^)	0.2	16.1	5.6	3.6

### Height-Diameter (*H*-*D*) modelling

*H*-*D* modelling was carried out in R environment (version 3.1 [66]), using the package “nlme” (version 3.1–117 [67]), which fits non-linear mixed-effects models by maximum likelihood (ML). We compared the performance of a large number of model forms available in the literature [39] using the model comparison tools available in package “lmfor” (version 1.1 [68]). Two-parameter Curtis’s function [36] showed the best fit while conforming to the assumptions of normality, homoscedasticity and lack of trend in the residuals:

$$H\left(D\right)=1.3+\frac{\alpha D}{{\left(1+D\right)}^{\beta }}$$ (1)

where 1.3m is the breast height at which diameters were measured and *α*,*β* are model parameters. Therefore, the general model can be expressed as:

$$h_{ijk}=f\left(\alpha ,\beta ,x_{ijk}\right)+e_{ijk},\;i=1,\ldots ,n_{jk},\;j=1,\ldots ,M_{k},\;k=1,\ldots ,K,$$ (2)

where *h*_*ijk*_ is the *H* of a tree *i* measured within plot *j* in cluster *k*, and *e*_*ijk*_ the model residuals. *f* is the non-linear function, Eq 1 in this case, (***α***,***β***) being its parameter vectors. ***x***_*ijk*_ is a vector of covariates used for predicting *H*, which was only *D* in the simplest case. Therefore, the case for the simple fixed-effects *H*-*D* model (**M1**) was defined by:

$$x_{ijk}=d_{ijk}$$ (3)

$$\alpha =\alpha_{0}$$

$$\beta =\beta_{0}$$

Then, we considered the possibility of accounting variability in these parameters due to the presence of 36 different species (*sp*), which may show a different *H*-*D* relation due to physiological differences and interspecific competition within the plant:) no more than plantsaying? (and e.ves a greater impression of high species mixture. (). a case study)this whole thing can community. The species of each *H*-measured tree (*sp*_*ijk*_) were therefore included in the covariate vector, as well as species-specific parameters (*α*_*sp*_,*β*_*sp*_). An additional term for *sp* was therefore added, obtaining a second fixed-effects *H*-*D* model (**M2**) as:

$$x_{ijk}=\left(d_{ijk},sp_{ijk}\right)$$ (4)

$$\alpha =\alpha_{0}+\alpha_{sp}$$

$$\beta =\beta_{0}+\beta_{sp}$$

Next, between-plot variability was accounted for by adding plot random effects (*a*_*j*_,*b*_*j*_) as an additional term in a mixed-effects model (**M3**) as:

$$x_{ijk}=\left(d_{ijk},sp_{ijk}\right)$$ (5)

$$\alpha =\alpha_{0}+\alpha_{sp}+a_{j}$$

$$\beta =\beta_{0}+\beta_{sp}+b_{j}$$

Finally, the last step was to consider the clustered sampling design followed after LDSF [62] (Fig 1B), by using nested levels of grouping for these random effects. The final mixed-effects model (**M4**) therefore included two random effects: a first (outer) level of grouping for clusters ($b_{k}^{\prime }$), and a second (inner) level for the plots contained within each cluster (*b*_*jk*_):

$$x_{ijk}=\left(d_{ijk},sp_{ijk}\right)$$ (6)

$$\alpha =\alpha_{0}+\alpha_{sp}+a_{jk}+a_{k}^{\prime }$$

$$\beta =\beta_{0}+\beta_{sp}+b_{jk}+b_{k}^{\prime }$$

To summarize, as a result of *H*-*D* modelling, we obtained the following *H* estimations for each tree:

- **M1**: *H* predicted from model based on *D* fixed effects (Eqs 2 and 3);
- **M2**: *H* predicted from model including *D* and species as fixed effects (Eqs 2 and 4);
- **M3**: *H* predicted from mixed-effects model including *D* and species as fixed effects and between-plot random effects (Eqs 2 and 5); and
- **M4**: *H* predicted from mixed-effects model including *D* and species as fixed effects and multilevel nested between-cluster and between-plot random effects (Eqs 2 and 6).

Accuracy of each model was assessed by computing the bias (mean difference of predicted and observed values) and root mean square error (RMSE) of model residuals, and expressing them in both absolute (m) and relative (%) units. Since effects were included as nested additive terms, increasing model complexity, whether the additions incorporated significant portions of explained variance was determined with likelihood ratio (LR) tests of their ML fits, using “lmtest” package [69]. Therefore, in LR tests a *χ*^2^ distribution was used to test the null hypothesis, which was the goodness of fit without the additional term (i.e., minus log-likelihood of the model of immediately decreasing complexity from **M4** to **M1**).

### Modelling steps

The general modelling setting described in the previous sub-section was subject to diagnose of model assumptions, which shaped the final models presented in results. According to Eq 3, the simple fixed-effects (*α*_0_,*β*_0_) model **M1** was fitted as:

$$h_{ijk}=1.3+\frac{\alpha_{0}d_{ijk}}{{\left(1+d_{ijk}\right)}^{\beta_{0}}}+e_{ijk}$$ (7)

This model obtained significant estimates for all parameters (see Table 2 in results). The model form chosen achieved a homogeneous distribution of residuals, which was specially challenging for larger trees, rendering other alternatives unreliable. Although **M1** was unbiased, it was considered that its precision could be improved by including additive effects from other covariates available. The first such covariate considered was the effect of species, since in highly mixed-species forest it was likely that some species may significantly differ from the main effects in Eq 7. Our strategy was to selected the species to be added as fixed effects on the grounds of statistical significance, based on two steps: (i) incorporating these effect for all species, as a means for selecting the significant ones, and (ii) using those selected ones in a final fixed-effects model predicting *H* from both *D* and species. Then, after obtaining unreliable intercept estimates when adding species effects in the numerator of the equation (*α*_*sp*_; Eq 4), it was decided to incorporate these fixed effects in the exponent of the denominator (*β*_*sp*_; Eq 4) only. For the first step to select significant species, we added a categorical variable describing all the *H*-measured species (*sp*_*ijk*_) as fixed effects:

$$h_{ijk}=1.3+\frac{\alpha_{0}d_{ijk}}{{\left(1+d_{ijk}\right)}^{\left(\beta_{0}+\beta_{sp}sp_{ijk}\right)}}+e_{ijk}$$ (8)

**Table 2:** Parameter estimates for the models M1-M4. *σ*: standard deviations. SE: standard errors. RMSE: root mean square error. *D*: tree diameter; *j*: plot; *k*: cluster. Levels of significance: **, <0.01; ***, <0.001.

	M1: Fixed-effects (Fixed = *D*)		M2: Fixed-effects (Fixed = *D* + species)		M3: Mixed-effects (Fixed = *D* + species; Random = plot)		M4: Mixed-effects (Fixed = *D* + species; Random = plot + cluster)	
Parameter^†^	Estimate	SE	Estimate	SE	Estimate	SE	Estimate	SE
*α*_0_	1.378***	0.161	1.281***	0.129	1.299***	0.120	1.290***	0.120
*β*_0_	0.484***	0.035	0.487***	0.030	0.491***	0.028	0.490***	0.028
*β*_*A*.*leiocarpus*_			-0.122***	0.009	-0.119***	0.010	-0.118***	0.010
*β*_*B*.*africana*_			-0.065***	0.013	-0.068***	0.014	-0.065***	0.014
*β*_*C*.*molle*_			-0.078**	0.024	-0.075***	0.022	-0.074***	0.022
$\sigma_{b_{j}},\sigma_{b_{jk}}$					0.041		0.034	
$\sigma_{b_{k}^{\prime }}$							0.023	
*σ*_*res*_	2.027		1.728		1.424		1.421	
RMSE (m)	2.022		1.717		1.516		1.549	
RMSE (%)	25.64		21.78		19.23		19.65	
Bias (m)	-8.77∙10^−5^		1.28∙10^−2^		3.45∙10^−2^		2.13∙10^−2^	
Bias (%)	-0.001		0.162		0.437		0.270	

^†^ see Eqs 7, 9, 11 and 12.

Considering significances at the 95% level, five were the species which obtained a significant estimate for the *β*_*sp*_ parameter (in order of significance): *Anogeissus leiocarpus*, *Burkea africana*, *Combretum molle*, *Prosopis africana* and *Pterocarpus erinaceus*. However, a model including these five species effect as dummy variables violated model assumptions, showing the influence of outliers in residuals, and presented an estimate for *P*. *erinaceus* with high standard error. Accordingly, this model was rejected and, by increasing the significance level to 99%, the number of species was reduced to three: *A*. *leiocarpus*, *B*. *africana* and *C*. *molle*. Therefore, these species were selected and added as dummy variables (denoted by the first letters of their genus and species: *Al*_*ijk*_; *Ba*_*ijk*_; *Cm*_*ijk*_), resulting in a simpler model **M2**:

$$h_{ijk}=1.3+\frac{\alpha_{0}d_{ijk}}{{\left(1+d_{ijk}\right)}^{\left(\beta_{0}+\beta_{Al}Al_{ijk}+\beta_{Ba}Ba_{ijk}+\beta_{Cm}Cm_{ijk}\right)}}+e_{ijk}$$ (9)

As in other cases, these effects were also initially tested to modify both the intercept (***α***) and the slope (***β***), however in the end they were only left in the slope due to lack of significance in the intercept.

The next step was to include the plot random effects into a mixed-effects model (**M3**). According to Eq 5, plot random effects (*a*_*j*_,*b*_*j*_) were incorporated from Eq 13 as:

$$h_{ijk}=1.3+\frac{\left(\alpha_{0}+a_{j}\right)d_{ijk}}{{\left(1+d_{ijk}\right)}^{\left(\beta_{0}+\beta_{Al}Al_{ijk}+\beta_{Ba}Ba_{ijk}+\beta_{Cm}Cm_{ijk}+b_{j}\right)}}+e_{ijk}$$ (10)

Although, when this model was fitted, the assumptions of independent and identically distributed random effects was not met. After detecting high correlation between the random effects *r*(*a*_*j*_,*b*_*j*_) = −0.997, it was decided to leave the random effects only in the exponent of the denominator (i.e. *a*_*j*_ = 0). Therefore, we obtained a simpler mixed-effects model accounting for plot effects (**M3**) which was finally used:

$$h_{ijk}=1.3+\frac{\alpha_{0}d_{ijk}}{{\left(1+d_{ijk}\right)}^{\left(\beta_{0}+\beta_{Al}Al_{ijk}+\beta_{Ba}Ba_{ijk}+\beta_{Cm}Cm_{ijk}+b_{j}\right)}}+e_{ijk}$$ (11)

**M3** was only useful in case that at least one height measurement was available for a given plot. There were, in fact, seven plots out of the total 152 plots containing trees, for which no height measurement was taken. In order to obtain the final *AGB* estimation based in both *D* and *H* (see next section below), the *H* prediction from **M2** was taken for those few plots.

Finally, the convenience of using a more complex multilevel mixed-effects (**M4**) accounting for both between-plots effects (*b*_*jk*_) nested within between-clusters effects ($b_{k}^{\prime }$) was also evaluated as:

$$h_{ijk}=1.3+\frac{\alpha_{0}d_{ijk}}{{\left(1+d_{ijk}\right)}^{\left(\beta_{0}+\beta_{Al}Al_{ijk}+\beta_{Ba}Ba_{ijk}+\beta_{Cm}Cm_{ijk}+b_{jk}+b_{k}^{\prime }\right)}}+e_{ijk}$$ (12)

As in the previous cases, the inclusion of these effects in both intercept and slope (Eq 6) was initially considered but dismissed after observing lack of statistical significance for the intercept. Similar restrictions to the use of **M3** apply for **M4** as well, and therefore the prediction of *H* from model **M2** had to be taken for the few plots where no tree heights were measured, with the intention of obtaining the corresponding final *AGB* estimation.

### Computation of aboveground biomass

We used pan-tropical allometric models from Chave et al. [24] for predicting *AGB*. The models are based on trees (*D* from 5 to 212 cm) harvested from a wide range of tropical climatic conditions and vegetation types (tropical forests, subtropical forests and woodland savannas). When *H* is available, the best-fit pan-tropical model for tree *AGB* (kg) is (Eq 4 in Chave et al. [24]):

$$AGB=0.06773\;{\left(\rho \cdot D^{2}\cdot H\right)}^{0.976}$$ (13)

which includes the effect of species-specific wood densities (*ρ*; g cm^–1^). In the absence of *H*, the following alternative model is recommended (Eq 7 in Chave et al. [24]):

$$AGB=\text{exp}\left[-1.803+0.976\left(\ln\left(\rho \right)-E\right)+2.673\;\ln\left(D\right)-0.0299{\left[\text{ln}\left(D\right)\right]}^{2}\right]$$ (14)

where *E* is a dimensionless measure of environmental stress based on temperature seasonality (*TS*), climatic water deficit (*CWD*) and precipitation seasonality (*PS*) (Eq 6b in Chave et al. [24]):

$$E=\left(0.178\cdot TS-0.938\cdot CWD-6.61\cdot PS\right)\cdot 10^{-3}$$ (15)

We computed *AGB* for each tree in the 0.1-ha plots using Eq 13 and different *H* predictions. *AGB* was also computed for median diameter trees in the 0.01-ha subplots using the measured *H*. Wood density values *ρ* were derived from Nygård and Elfving [70] and online databases [25,71]. They were assigned to each tree from the closest taxonomic level available [44,72]. 97.5% of stems had *ρ* data available from species level and 100% from genus level. Averaged $\bar{\rho }$ values were used whenever there were multiple values for a same species. On the other hand, *AGB* based on *D* only was computed using Eq 8. The value of *E* (0.701) was extracted from the global gridded dataset (2.5 arc sec resolution) provided by Chave et al. [24], and given to the entire study area. To finalize, in order to compute plot-level *AGB* predictions (Mg ha^–1^), we assigned a hectare expansion factor (HEF) for each stem, according to the size of the plot/subplot where it was measured (i.e. HEF = 10 for 0.1-ha plots, and HEF = 25 for the four 0.01-ha subplots).

As a result of all the above-mentioned options for *H*-*D* modelling, we also obtained a similar list of different plot-level *AGB* predictions:

- **B0**: *AGB* obtained from measured *D* only (Eq 14);
- **B1**: *AGB* obtained from measured *D* and predicted *H* (Eq 13), using **M1** model with *D* fixed effects (Eqs 2 and 3);
- **B2**: *AGB* obtained from measured *D* and predicted *H* (Eq 13), using **M2** model with *D* and species fixed effects (Eqs 2 and 4);
- **B3**: *AGB* obtained from measured *D* and predicted *H* (Eq 13), using **M3** model with *D*, species fixed effects and between-plot random effects (Eqs 2 and 5);
- **B4**: *AGB* obtained from measured *D* and predicted *H* (Eq 13), using **M4** model with *D*, species fixed effects and multilevel nested between-cluster and between-plot random effects (Eqs 2 and 6);

### Effects of environmental stress factor (*E*) and baseline determination

It is worth noting that the *AGB* allometry without *H* as predictor in Eq 14 assumes a pan-tropical *H*-*D* model that depends on *E* (Eq 6a in Chave et al. [24]):

$$\ln\left(H\right)=0.893-E+0.760\;\ln\left(D\right)-0.0340{\left[\text{ln}\left(D\right)\right]}^{2}$$ (16)

**Fig 3:**
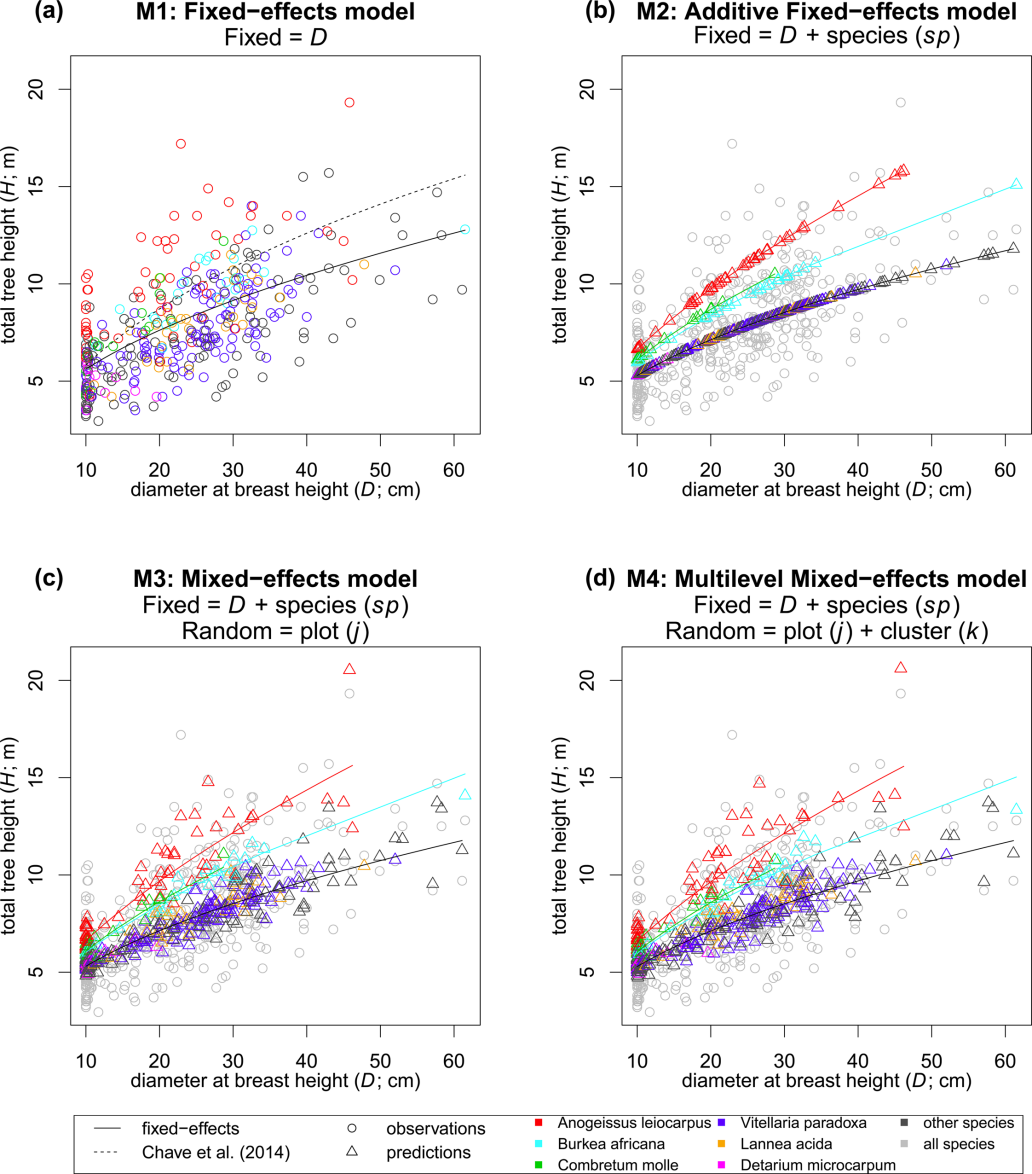
Model fit plots. Effects were included as nested additive terms, increasing complexity: **(a)** fixed-effects *H*-*D* model; **(b)** adding species fixed effects; **(c)** adding plot random effects; and **(d)** adding cluster random effects. Predictions have been omitted from (a), since they are known to follow the fixed-effects model (solid line), so that the species distribution in the training data can be observed more clearly in this plot.

In other words, in a hypothetical case of empirical *H*-*D* data fitting exactly to this relation, there would be no difference between Eqs 13 and 14 for predicting *AGB*. In the context of the present article, we name this model **M0**, as the *H*-*D* model that corresponds to the *AGB* predictions obtained by **B0** (for comparison to our own models **M1**-**M4**, the pan-tropical model has been incorporated to Fig 3A, see results). We considered that such hypothetical case could be regarded as a ‘baseline’ (assumed most accurate, in absence of destructive sampling of *AGB*) to which all other alternatives could be compared. For that purpose, using our empirical *H*-*D* data, we solved *E* from Eq 16. The result was an alternative *E* ‘calibrated’ to the specific local conditions defined by the empirical *H*-*D* relations measured in the field, which can be applied directly in Eq 14. The calibration was carried out by including the exponential form of Eq 16 in function “nls” [66], obtaining the adjusted value for *E* which best fits to the measured *H*-*D* data. In the context of the present article, these are the baseline model (M0*) and its subsequent *AGB* predictions (B0*).

### Assessing statistical differences among alternatives

We were especially interested in detecting whether the choice of method can actually lead to bias in *AGB* estimation, which is a conclusion that can be deducted by simply comparing methods. In the absence of *AGB* data obtained by destructive sampling which would allow an independent validation of the final *AGB* estimations, we considered that accounting for differences between the modelling approaches can provide an idea on how differences in *H*-*D* modelling can propagate to the final *AGB* predictions [11,20]. After obtaining all the alternative *AGB* predictions (**B0**-**B4** and the baseline B0*), we tested whether they were significantly different, or otherwise the *AGB* allometry and *H*-*D* modelling approach does not significantly affect the final outcome. With that intention, we computed pair-wise differences (*diff*) between all plausible combinations of the alternatives. Since normality could not be assumed for all groups, the hypothesis that two alternatives were significantly different was tested using paired Wilcoxon signed rank tests (R function “wilcox.test”).

## Results

### *H*-*D* modelling

Fig 3 shows the fit for the final *H*-*D* models, whereas Table 2 details their estimates and summarizes their performance in terms of accuracy and bias. Fig 3A also includes the fit that would correspond to model **M0** (dashed line), which is the assumed *H*-*D* relation (Eq 16) for the *AGB* prediction using only *D* as predictor (**B0**). Table 3 shows the results of model comparison by LR test.

**Table 3:** Likelihood ratio tests comparing nested models. -LogLik.:—log of likelihood; *D*: tree diameter. Levels of significance: **, <0.01; ***, <0.001.

		*χ*^2^		
	-LogLik.	M2	M3	M4
**M1**: Fixed-effects	855.65	131.85***	172.65***	180.96***
**M2**: Fixed-effects (*D* + species)	789.73		40.80***	49.11***
**M3**: Mixed-effects (*D* + species + plot)	769.33			8.31**
**M4**: Mixed-effects (*D* + species + plot+ cluster)	765.17			

**Fig 4:**
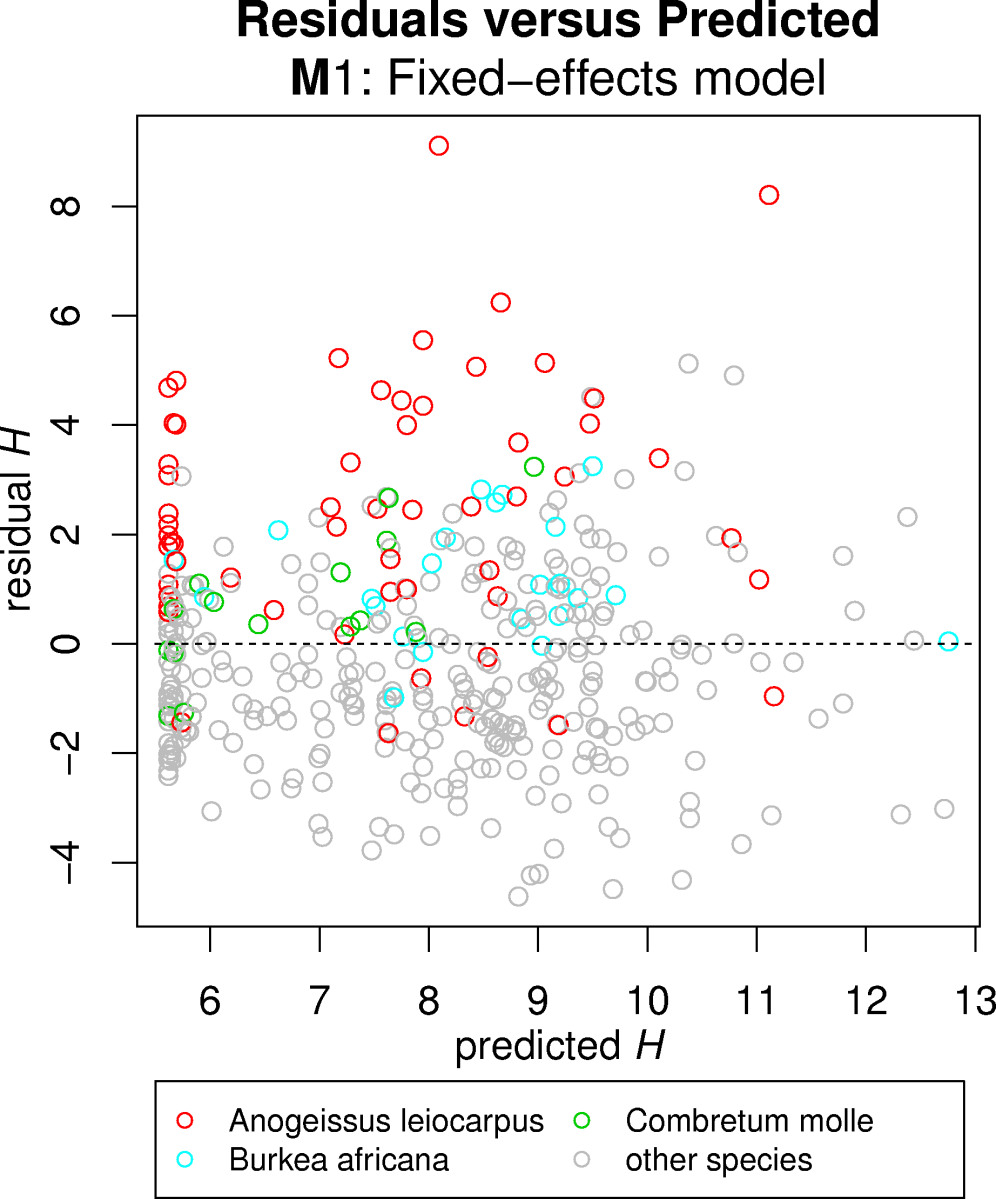
Plot of residuals versus predictions for fixed-effects *H*-*D* model (M1). Species which were found to significantly include additional effects are highlighted.

Nested additive terms were successively included so that models were increasing in complexity. **M1** was accepted as a valid and unbiased *H*-*D* model, although its RMSE of 25.64% was deemed quite high (Table 3), and therefore we considered the possibility of including additional effects. Observing the model fit against the training data, it became apparent that some species (Fig 3A) stand out from the general *H*-*D* relation in this forest community. When individual species were highlighted in residual versus prediction plots, some of those residuals appeared to be highly biased for few individual species (Fig 4). It therefore seemed to exist an important species effect in the *H*-*D* relation (Eq 4), although there was a challenge in defining for which species a modification in the model was actually necessary. Using the two-step approach to determining significant species effects, as detailed in sub-section “modelling steps”, the following species were finally selected for species effects: *A*. *leiocarpus*, *B*. *africana* and *C*. *molle* (Fig 4). Although the significance for the latter one was weak (Table 2), it was accepted as the standard error of its estimate was acceptable. Model **M2** (Fig 3B) explained a substantially higher proportion of variance than **M1**, achieving a reduction of the RMSE down to 21.78% (Table 2). Results from the LR test demonstrated the significance of including these additional terms in the model (Table 3).

The next increase in model complexity was the inclusion of plot random effects. Model **M3** delivers predictions (triangular symbols in Fig 3C) that differ from the fixed part of the model (solid lines in Fig 3C) according to ratios of variances within-plots and between-plots ($\sigma_{b_{j}}$). **M3** was very successful in terms of model fit, since all parameter estimates were significant (Table 2), with residuals from Eq 11 showing normality, heteroscedasticity and independence of predictors and random effects, and also lacking dependencies (correlations) among random effects (not the case for Eq 10, which was therefore dismissed). Compared to **M2**, **M3** obtained a considerable increase in the amount of explained variance and lower RMSE (Table 2). LR test demonstrated the significance of incorporating the between-plot variability as random effects into the previous fixed-effects model **M2** (Table 3), and it was therefore accepted the inclusion of this additional term (*b*_*j*_).

Furthermore, as detailed in Eq 12, a final multilevel mixed-effects (**M4**) including nested levels for plot and clusters effects, was obtained. In the case of model **M4**, final predictions (triangular symbols in Fig 4C) depend on ratios of within-groups variances with respect to between-clusters variance ($\sigma_{b_{k}^{\prime }}$) and variances between-plots at each cluster ($\sigma_{b_{jk}}$). Diagnosis of the resulting model **M4** (Fig 3D) was positive, although the amount of explained variance was very similar to the other simpler mixed-effects model **M3** (Table 2). A very surprising outcome was to observe a slight increase in the RMSE of **M4** with respect to **M3**. Nonetheless, the LR ratio test between these two models was still significant at a 95% level (Table 3), and therefore the incorporation of this nested additional random effect between clusters could, in principle, be accepted.

### Comparison of *AGB* estimates

**Fig 5:**
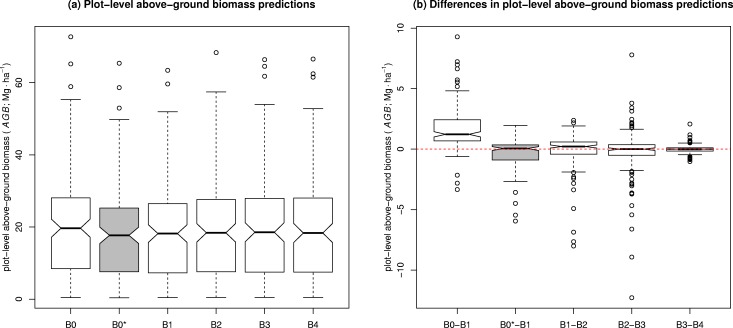
**(a) Above-ground biomass (Mg∙ha**^**-1**^**) predictions at plot-level obtained by different methods and (b) their absolute differences**. For simplicity, differences are shown sequentially, comparing methods which increase the complexity of prediction successively. **B0**: Eq 14; **B1**: Eqs 13 and 7; **B2**: Eqs 13 and 9; **B3**: Eqs 13 and 11; **B4** Eqs 13 and 12; B0*: Eqs 14 and 16.

Five different plot-level *AGB* predictions (**B0**-**B4**) were obtained, each corresponding to one *H*-*D* model alternative (**M0**-**M4**). (Fig 5A) compares their box-whisker plots and Table 4 includes their summary statistics. Table 4 also presents a matrix of all combinations for pair-wise differences (*diff*) whereas, for simplicity, the box-whisker plots selected for Fig 5B only compare differences between options of successively increasing complexity: **B0** versus **B1**; **B1** versus **B2**; **B2** versus **B3**; and **B3** versus **B4**. We used actual Mg∙ha^-1^ units to help the reader in reflecting on the practical significance of absolute *AGB* differences. Positive *diff* values denote underestimation with respect to the simpler option, and negative corresponds to overestimation (i.e., *diff* is rows minus columns in Table 4). Medians and interquartile ranges (IQR) for *diff* are reported, because the distribution of some of the differences was extremely asymmetric. Table 4 also includes a matrix of significance results for the Wilcoxon signed rank tests for those differences. The null hypothesis for those tests was that differences between two groups equal zero (H_0_: *diff* = 0).

**Table 4:** Comparison of above-ground biomass (*AGB*) estimates including summary of plot-level $\bar{AGB}$, pair-wise differences among methods (*diff*; row minus column), and significances yielded by paired Wilcoxon signed rank tests (H_0_: *diff* = 0). Levels of significance: ^NS^, not significant; ^•^, <0.1; *, <0.05;***, < .001. SD: standard deviation. IQR: inter-quartile range. *AGB*: above-ground biomass; *D*: tree diameter; *H*: tree height; *sp*: species; *j*: plot; *k*: cluster; *E*: environmental stress. **B0**: Eq 14; **B1**: Eqs 13 and 7; **B2**: Eqs 13 and 9; **B3**: Eqs 13 and 11; **B4** Eqs 13 and 12; B0*: Eqs 14 and 16.

	$\bar{AGB}$ (SD) (Mg ha^-1^)	*diff*: median (IQR) (Mg ha^-1^)					*p-value*				
		B1	B2	B3	B4	B0*	B1	B2	B3	B4	B0*
**B0**: *AGB* = *f*(*D*)	21.32 (14.47)	1.226 (1.74)	1.451 (2.10)	1.199 (2.35)	1.075 (2.30)	2.148 (1.47)	< .001***	< .001***	< .001***	< .001***	< .001***
**B1**: *AGB* = *f*(*D*,*H* = *f*(*D*))	19.49 (13.47)		0.218 (0.98)	0.117 (1.30)	0.021 (1.41)	0.331 (1.19)		.065^•^	.909^NS^	.786^NS^	.042*
**B2**: *AGB* = *f*(*D*,*H* = *f*(*D*,*sp*))	19.53 (13.67)			-0.013 (0.86)	-0.014 (0.92)	0.423 (1.67)			.634^NS^	.271^NS^	.050^•^
**B3**: *AGB* = *f*(*D*,*H* = *f*(*D*,*sp*,*j*))	19.72 (14.10)				-0.002 (0.26)	1.669 (0.86)				.239^NS^	.031*
**B4**: *AGB* = *f*(*D*,*H* = *f*(*D*,*sp*,*j*,*k*))	19.72 (14.06)					0.857 (3.14)					.043*
B0*: *AGB* = *f*(*D*,*E* = *f*(*D*,*H*))	19.17 (13.01)										

Although some of the differences seem negligible overall (Fig 5A), they may also be systematic and therefore lead to bias in *AGB* estimation (Fig 5B). Taking into account the worst case scenario (**B0** versus **B1** or **B2**), 95% of differences observed in plot-level predictions were reaching roughly 5.5 Mg∙ha^-1^ at most. However, *AGB* predictions from Eq 8 may be biased, since differences between option **B0** and the rest of alternatives were all significantly different than zero (Table 4). For instance, 82.3% of **B0 –B1** differences were above zero, which showed that using Eq 7 makes predictions systematically below those obtained by Eq 8, however with some exceptions.

This was not the case for the rest of contrasts carried out, which were largely non-significant, in spite of the significances found in the final fit of the *H*-*D* models (Table 3). Only the inclusion of species effects could be considered to significantly affect the final *AGB* predictions, although only considered under a 90% level of significance (*p-value* = 0.065; Table 4). Differences in the *AGB* predictions between approaches using fixed-effects or mixed-effects *H*-*D* models could be considered negligible, although it may be worth noting the presence of numerous outliers among the plot-level *AGB* differences obtained when the additional term incorporated were the random effects **B2 –B3** (Fig 5B).

### Effect of environmental stress factor (*E*)

**Fig 6:**
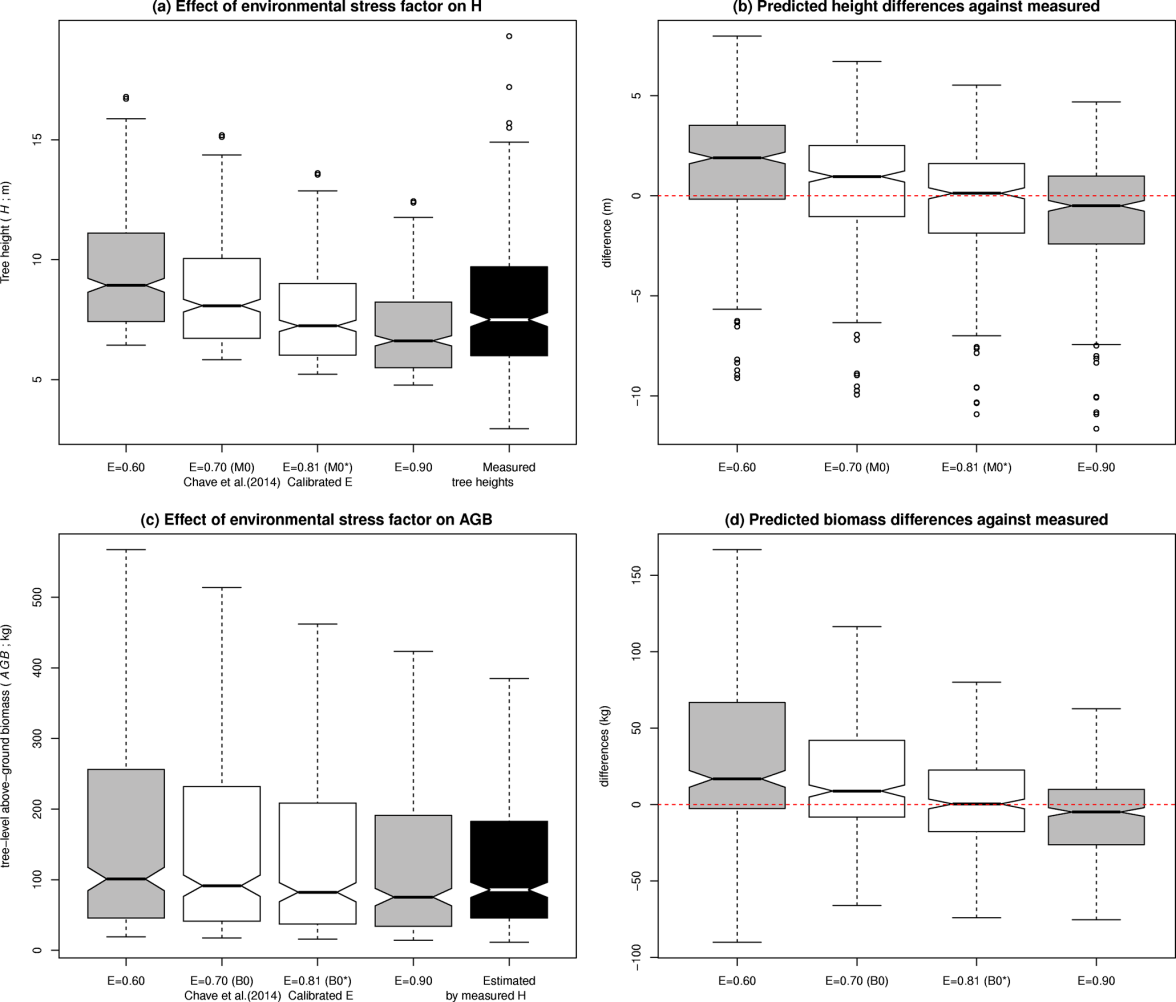
Sensitivity of tree height (*H*) and tree-level above-ground biomass (*AGB*) predictions to changes in the environmental stress parameter (*E*). **(a)** Tree *H* predictions by Eq 16, including (white colour) those obtained by the value of *E* = 0.7 extracted from Chave et al. [24] (**B0**; Fig 3A) and the calibrated baseline value (B0*; Table 5), and compared with those measured in the field (black colour). **(b)** Pair-wise differences of those *H* predictions against measured values. **(c)** Tree-level *AGB* predictions, also including **B0** and B0* (white colour), and compared with the *AGB* predictions obtained directly from field measurements, applying the corresponding Eq 13 (black colour). **(d)** Pair-wise differences of those *AGB* predictions and those obtained directly from field measurements. Outliers have been omitted from (c) and (d) to improve the clarity of figures.

Since the *AGB* estimations not using *H* as predictor in the allometry (Eq 14; **B0**), were notably different from all those including a *H*-*D* model, we considered it relevant to further investigate the reasons for what seemed an overestimation in **B0**. In order to investigate the sensitivity of the *AGB* estimations to changes in the *E* parameter, we employed Eq 16, which tells us the *H* that on average corresponds each *D* under the environmental stress conditions defined by a given value of *E*. In Fig 3A, the dashed line depicts that model for the exact case of *E* = 0.70 applied in this study site (**M0**). Fig 6 shows how changing values of *E* within a range of 0.6–0.9 affects the prediction of *H* from *D*, and how this propagates to *AGB* predictions at the tree level (**B0**).

As explained in the sub-section about “assessing statistical differences among alternatives”, we determined baseline *AGB* predictions as those for the value of *E* which secured the best fit of Eq 16 to the empirical *H*-*D* data (M0*). This ‘calibrated’ value was found to be *E* = 0.81 (Table 5), and its subsequent *AGB* predictions (B0*; Eq 14) were unbiased with respect to those obtained directly from the field *H*-*D* measurements, via Eq 13 (Fig 6D). The inclusion of the red dashed line within boxplots’ notches in Fig 6B and 6D, are therefore also an indication of whether they are significantly equal to the measured values, whereas its exclusion from within those notches signify a possibility for bias. Also, to help the reader in reflecting on the absolute *AGB* differences, we incorporated plot-level aggregates of B0* to Table 4 and Fig 5A. Moreover, Fig 5B also includes B0*–B1 differences, which can be directly compared to **B0 –B1**, showing the effect of calibrating *E* for obtaining the best fit to Eq 16. All by-group differences and their significances have also been added to Table 4. Differences between the baseline (B0*) and *H*-*D* modelling approaches were statistically significant, although significance levels were much lower than those for the approach using *D* only (**B0**).

**Table 5:** Baseline determination (*E* ‘calibration’) (M0*). *σ*: standard deviations. SE: standard error. RMSE: root mean square error. Level of significance: ***, <0.001.

	M0*: Calibration of *E*	
Parameter^†^	Estimate	SE
*E*	0.808***	0.018
*σ*_*res*_	2.841	
RMSE (m)	2.837	
RMSE (%)	35.94	
Bias (m)	-0.279	
Bias (%)	-3.535	

## Discussion

In the framework of a project quantifying carbon stocks and inventorying fuel wood resources, this particular research aimed at detecting the effect of *H*-*D* modelling in *AGB* estimation using many of the options available in the literature. Chave et al. [24] recommended to include *H* as a predictor in *AGB* allometry, and preferably develop locally-fitted *H*-*D* models for each study. The cluster sampling design employed in our field data collection and the high number of species present in the study area led us to an *a priori* assumption that the correct approach should be a multilevel mixed-effects model designating the hierarchical levels of sampling as random effects, and also including fixed effects accounting for species influence. However, as detailed in the results for *H*-*D* modelling, the development of such a complex model requires some skill in carefully checking that modelling assumptions are met and no critical dependencies are left among random effects, residuals, etc. [50]. On the other hand, Mehtätalo et al. [39] found no critical differences among *H*-*D* modelling approaches, while Feldpausch et al. [47] recommended to include plot-level random effects in tropical *H*-*D* allometric models. The key question for us was: does the *H*-*D* modelling approach actually matter to the final purpose of *AGB* estimation, or could otherwise the simplest option work out a proper prediction for *AGB*?

### Multilevel versus single-level random effects

Considering the final *AGB* estimations, differences between the *H*-*D* models including nested multilevel random effects (**B4**) and the single-level plot random effects (**B3**) were negligible and probably lacked importance in practice. Differences were only marginal, dispersing narrowly around zero (Fig 5B), within an approximate 95% confidence interval of– 0.002 ± 0.50 Mg∙ha^-1^. Therefore, in spite that the inclusion of nested multilevel random effects was conceptually the most appropriate approach for the sampling design based on LDSF [61,62], adding such complexity to the *H*-*D* model may not matter much in practice, after all.

Furthermore, although the LR tests found the inclusion of cluster-plus-plot random effects significant compared to plot-only (Table 3), the significance level was lower than other contrasts’. It was also observed that **M4** increased the RMSE with respect to **M3**, which may be a sign of overfitting for the multilevel *H*-*D* model. In conclusion, since the complexity of the model was increased without substantially improving its precision, we decided that model **M3** with single level of grouping for random effects was more convenient in practice. However, this result may reflect an absence of significant landscape-level differences or gradients influencing the *H*-*D* relation at our study area. This may be the case since, for example, the entire study area shared a same value of environmental stress (*E*) (sensu Chave et al. [24]). The inclusion of multilevel random effect could, however, be relevant in studies following LDSF inventory design over larger areas, or dealing with more heterogeneous landscapes or latitudinal/altitudinal gradients.

### Mixed-effects versus fixed-effects models

Mixed-effects models outperformed fixed-effects ones in terms of LR tests and accuracies. However, the plot random effects (*b*_*j*_) are only useful in plots where at least one tree has been measured, providing a calibrated prediction [73]. In turn, the advantage is that this same model could be applied on additional field plots from this same study area if acquiring a higher sample size was considered necessary [32,46].

Although results in *H*-*D* modelling suggested the importance of the mixed-effects model **M3** with regards to the final plot-level *AGB* estimations (Table 4), we found no substantial differences between the models that included random effects (**B3**-**B4**) and those which did not (**B1**-**B2**). This does not mean that *AGB* differences between these approaches are not important, but that they are not systematic. As mentioned, in the absence of data for external validation, we cannot reflect on the accuracy of each approach, but only study differences in means and infer that they could indicate the presence of bias. In the light of our results, there are no systematic differences between the *AGB* estimations based on the fixed-effects and mixed-effects models, and therefore omitting the random effects would not lead to bias in the *AGB* estimation for the study area. This is, however, not an unexpected outcome, since it demonstrates that the random effects are actually random. Nonetheless, Fig 5B shows the presence of a large number of outliers in **B2 –B3**. This may mean that the mixed-effects model is mending the prediction for few given plots, which were probably needing it, possibly having characteristics that makes them differ slightly from others, such as soil [47], site productivity, or relative stand density [22]. Differences between **B2** –**B3** show that *AGB* discrepancies were enclosed within– 0.013 ± 1.69 Mg∙ha^-1^ (Table 4). This suggests that, although the fixed-effects model would by itself lead to an unbiased *AGB* estimation for the entire study area, the mixed-effect models may correct some large errors which may be relevant at some plots, better reflecting the spatial distribution of *AGB*. The most conservative choice is therefore to use mixed-effects modelling to avoid error at such areas, and therefore **B3** is our recommended option among those compared.

### *H*-*D* modelling with species effect versus *D*-only

The effect of species observed in the *H*-*D* model showed that only few species had a slightly different relation than others (Fig 4). These may be species that dominate the top of the canopy and grow taller for a same *D* than other species do [74]. Availability of sufficient sample size for a given species may also critical for finding significant differences in its *H*-*D* relation, and therefore the species that were finally found to significantly differ from the others–*A*. *leicocarpus*, *B*. *africana* and *C*. *molle*–also show some of the highest frequencies in the study area (Fig 1). There was, however, no univocal relation between sample size and significance, since not all species with large sample sizes were found to significantly affect the *H*-*D* relation (36 species tested). In practice this shows the reliability of the method for identifying significant species effects, but it also means that we cannot statistically assure whether a different *H*-*D* relation should apply the rest of species, as probably many more among the non-significant ones do. In turn, since *A*. *leicocarpus*, *B*. *africana* and *C*. *molle* collectively account for almost 40% of the total number of stems (Fig 1), their effect on the final *AGB* computations is greater. As a result, not only including the additional term for species was found to be significant for *H* prediction, but the derived plot-level *AGB* estimation was significantly different (**B1** versus **B2**; Table 4). These differences reached 0.218 ± 1.69 Mg∙ha^-1^, which may be relevant specially if considering that there is an overall bias leading to overestimation if no species information is included. All these significances found in the species fixed effects showed the importance of accounting for species-wise differences in *H*-*D* modelling of highly-mixed tropical forests. Therefore, the effects by species were demonstrably the most relevant we found among those considered. However the inclusion of random effects, although being systematic and of lesser importance, may also lead to improvements in some given plots.

Topics related to the effect of species in allometry and methods dealing with species mixture in *AGB* estimations are of current interest in the research literature [22,23,75]. Typical approach is to derive separate *H*-*D* allometry for each species (e.g., [39–42]), although this may be unpractical in highly mixed forests [27]. The alternative is to use pan-tropical models [24], in which it is commonly assumed that the effect of species in biomass estimations is implicitly included when considering species-specific *ρ* in the *AGB* allometry (e.g., [29]). However, even though *ρ* was included in **B1** (Eq 7), we still observed a weak significance in its plot-level differences against the *AGB* predictions of **B2**, which made use of the *H*-*D* model accounting from species effect (Table 4). This may indicate a possibility for bias in *AGB* predictions using a pan-tropical *AGB* model if the species effect is not accounted for in the *H*-*D* model. In our study, we were even surprised to find that the fixed species effect was more relevant than the random plot effect, in terms of added explained variance. Accordingly, we suggest to include additional terms accounting for species effects in the *H*-*D* models. In this article, we presented a two-step replicable methodology to tackle with the presence of multiple species in a same study area. In the first step, species was added as a fixed-effect factor only with the purpose to identify the significant species, which were added as separate dummy variables in the second step and final model. We suggest that this method can be easily replicable in other highly mixed-species forest areas.

### *AGB* allometry using *D*-only versus *D* and *H*

The largest differences were found between the *AGB* estimations obtained from the allometry involving only *D* (**B0**; Eq 8), and those including both as predictors (**B1**-**B4**; Eq 7). Results were very significant, with clearly biased differences which reached levels within a 95% confidence interval of 1.451 ± 4.12 Mg∙ha^-1^ (Table 4). This result was not surprising, since it is convergent with conclusions drawn by Chave et al. [11, 24], that best approach is to derive local *H*-*D* allometry for each study. Some readers may think of a plausible fifth alternative to those contrasted in this article: apply a published *H*-*D* model and use those *H* predictions in Eq 13. It is noteworthy to mention that we also tested *H*-*D* models published by Feldpausch et al. [27] and Banin et al. [74], only to observe that such approach would dramatically overestimate *H* and hence *AGB* for our study area. The reason is that those models have been fitted in very different kind of forests and conditions than those present at this study site. The fact that they are unbiased over the extent at which they were adjusted (pan-tropical, continental or regional) does not prevent that they can lead to bias if applied locally, like it was for our case. In contrast, the flexibility provided by the *E* parameter in Chave et al.’s [24] models allows modifying the intercept for each case study, allowing locally-unbiased implementation of the model.

### Sensitivity to the environmental stress parameter

We also though that inaccurate assumptions in *E* may be conditioning the *AGB* predictions using only *D*, and decided to further investigate their sensitivity to *E*. The resulting test demonstrated the high sensitivity of the *AGB* predictions to *E*, and since it affects to the mean *H* and *AGB*, any uncertainty in *E* induce bias rather than random errors (Fig 6C). This begged the following question: what value of *E* would provide correct mean *H* for our study area? Then we developed the ‘calibrated-*E*’ approach and its derived *AGB* predictions. Although they were still significantly different than the predictions yielded by *H*-*D* modelling, those differences were no longer systematic, serving as a baseline for our study. The magnitude of change in was higher than 0.1, which leads to relevant changes in the predictions, since Eq 14 is critically sensitive to the value chosen for describing the environmental stress (Fig 6). In our case, the values of *E* that would provide correct mean *AGB* can be found from Chave et al. [24] less than 100 km northward from our study area. Since our study site was not large enough to cover areas with different values for *E*, we are unable to detect a systematic error of *E*. However, the overestimation effect in the *AGB* allometry predicting from *D* only has also been observed by other authors [18,20,27], while we found no reports accounting for an underestimation of *AGB* when allometry lacks *H* as predictor. There is therefore a possibility that *E* may be more easily underestimated than overestimated, which needs to be further investigated. We postulate that there may be a need for including causes for environmental stress other than those included in the current definition of *E* (*TS*, *CWD* and *PS*; Eq 9) which may induce an increase in the ‘real *E*’, such as soil conditions and natural or human-induced forest disturbance. It may also be the case that underestimation is caused by measurement error of the parameters *TS*, *CWD* or *PS*, which should be object of further investigation. To derive their pan-tropical allometry, Chave et al. [24] employed data from old-growth and secondary forests, excluding plantations and agroforestry systems. Adding such components, could extend the applicability of their equations in tropical managed forests with high degrees of human intervention. This would bring an opportunity since perhaps such calibration could be carried out with a lower field effort in *H* measurements, although that would be only a hypothesis to be corroborated by further research.

Results showed that the alternative B0*, which is very simple in its implementation, can result in *AGB* predictions not systematically differing from those obtained by a separate *H*-*D* allometry, and therefore unbiased with respect to them. This indicates that the only reason for finding significant differences between **B0** and **B1** was the choice of model form. It must be recalled that the fixed-effects modelling procedure for calculating **B1** also included a comparison of different model forms, selecting the one which best fits to the empirical *H*-*D* data. The comparison is therefore unfair, and there are clear advantages in using a pan-tropical model. However, it could be suggested that an alternative version for Eq 16 which would allow changes in *E* to modify the slope, and not just the intercept, could yield more flexibility in model form, and perhaps the ‘calibrated-*E*’ approach would be more efficient as well.

## Conclusions and Suggestions for Future Research

In the light of these results, the modelling approach followed in some cases affected the final *AGB* estimation significantly, although differences were also negligible for some of the alternatives considered. The most important difference found was given by the additive term which accounted for species effect. Therefore, we recommend to include species as fixed effects in future allometry, as a simpler and more practical approach than obtaining models for each species in forests with high degree of mixture. The presented two-step methodology described for selecting species with significant effects, discriminating them from those that followed the general fixed-effects model, was successful in improving the accuracy of the *H* prediction, and proved relevant for the subsequent *AGB* estimation. We therefore suggest its replication in the similar studies. Further research could also test the same approach on *AGB* allometry, addressing the effect of species variation directly. Our final choice was the *H*-*D* mixed-effects model (**B3**), which accounts for the species but also for the plot random effects reflecting site factors such as soil properties and degree of disturbance. It should be noted that the practical use that we gave to the mixed-effects modelling would not be, however, applicable to the case of *AGB* modelling. The reason is that destructive sampling would be needed at every new field plot for calibrating its effect, which would be neither practical nor desirable.

We observed that *AGB* predictions without *H* were very sensitive to the chosen *E*, which represents the environmental stress of a given study site. We postulate that *E* may be systematically underestimated in forest areas with human intervention, although that extreme cannot be tested with our experimental design. Further research could investigate factors affecting *E*, considering the possibility of including an additional component for disturbance in Eq 15. Overall, factors most affecting the final *AGB* estimates were, in order of decreasing influence: the choice *E* (when using no *H*-*D* model), species variation, and plot-level variation. Cluster-level variation had lower influence, which may be sign of homogeneity within the given study area. Consequently, we concluded that alternatives rank in this order from the most desirable to the least accurate: (1) using mixed-effects *H*-*D* models accounting for species and plot-level variation; (2) using local fixed-effects *H*-*D* models; and (3) obtaining the *AGB* from *D* data only.

## References

[1] Cuny HE, Rathgeber CBK, Frank D, Fonti P, Mäkinen H, Prislan P, et al Woody biomass production lags stem-girth increase by over one month in coniferous forests. Nature Plants 2015 10/26;1:15160 doi: 10.1038/nplants.2015.160 27251531 10.1038/nplants.2015.160

[2] Eggleston HS, Buendia L, Miwa K, Ngara T, Tanabe K. IPCC Guidelines for National Greenhouse Gas Inventories Institute for Global Environmental Strategies 2006.

[3] UNFCCC—United Nations Framework Convention on Climate Change. Warsaw Framework for REDD+.; 2014.

[4] Clark DB, Kellner JR. Tropical forest biomass estimation and the fallacy of misplaced concreteness. Journal of Vegetation Science 2012;23(6):1191–1196.

[5] Sileshi GW. A critical review of forest biomass estimation models, common mistakes and corrective measures. For Ecol Manage 2014 10/1;329:237–254.

[6] Iais PC, Bombelli A, Williams M, Piao SL, Chave J, Ryan CM, et al The carbon balance of Africa: Synthesis of recent research studies. Philos Trans R Soc A Math Phys Eng Sci 2011;369(1943):2038–2057. 10.1098/rsta.2010.0328 21502175

[7] Valentini R, Arneth A, Bombelli A, Castaldi S, Cazzolla Gatti R, Chevallier F, et al A full greenhouse gases budget of africa: Synthesis, uncertainties, and vulnerabilities. Biogeosciences 2014;11(2):381–407.

[8] Sexton JO, Noojipady P, Song X, Feng M, Song D, Kim D, et al Conservation policy and the measurement of forests. Nature Clim Change 2015 10/05;advance online publication.

[9] Tesfaye MA, Bravo-Oviedo A, Bravo F, Ruiz-Peinado R. Aboveground biomass equations for sustainable production of fuelwood in a native dry tropical afro-montane forest of Ethiopia. Ann For Sci 2015:1–13.

[10] Nelson BW, Mesquita R, Pereira JLG, Garcia Aquino De Souza S, Teixeira Batista G, Bovino Couto L. Allometric regressions for improved estimate of secondary forest biomass in the central Amazon. For Ecol Manage 1999;117(1–3):149–167.

[11] Chave J, Andalo C, Brown S, Cairns MA, Chambers JQ, Eamus D, et al Tree allometry and improved estimation of carbon stocks and balance in tropical forests. Oecologia 2005;145(1):87–99. 15971085 10.1007/s00442-005-0100-x

[12] Basuki TM, van Laake PE, Skidmore AK, Hussin YA. Allometric equations for estimating the above-ground biomass in tropical lowland Dipterocarp forests. For Ecol Manage 2009;257(8):1684–1694.

[13] Sawadogo L, Savadogo P, Tiveau D, Dayamba SD, Zida D, Nouvellet Y, et al Allometric prediction of above-ground biomass of eleven woody tree species in the Sudanian savanna-woodland of West Africa. Journal of Forestry Research 2010;21(4):475–481.

[14] Ribeiro SC, Fehrmann L, Soares CPB, Jacovine LAG, Kleinn C, de Oliveira Gaspar R. Above- and belowground biomass in a Brazilian Cerrado. For Ecol Manage 2011;262(3):491–499.

[15] Kuyah S, Dietz J, Muthuri C, Jamnadass R, Mwangi P, Coe R, et al Allometric equations for estimating biomass in agricultural landscapes: I. Aboveground biomass. Agriculture, Ecosystems and Environment 2012;158:216–224.

[16] Vieilledent G, Vaudry R, Andriamanohisoa SFD, Rakotonarivo OS, Randrianasolo HZ, Razafindrabe HN, et al A universal approach to estimate biomass and carbon stock in tropical forests using generic allometric models. Ecol Appl 2012;22(2):572–583. 22611855 10.1890/11-0039.1

[17] Fayolle A, Doucet J-, Gillet J-, Bourland N, Lejeune P. Tree allometry in Central Africa: Testing the validity of pantropical multi-species allometric equations for estimating biomass and carbon stocks. For Ecol Manage 2013;305:29–37.

[18] Marshall AR, Willcock S, Platts PJ, Lovett JC, Balmford A, Burgess ND, et al Measuring and modelling above-ground carbon and tree allometry along a tropical elevation gradient. Biol Conserv 2012;154:20–33.

[19] Chave J, Condit R, Aguilar S, Hernandez A, Lao S, Perez R. Error propagation and scaling for tropical forest biomass estimates. Philosophical Transactions of the Royal Society of London B: Biological Sciences 2004 The Royal Society;359(1443):409–420. 15212093 10.1098/rstb.2003.1425 PMC1693335

[20] Molto Q, Rossi V, Blanc L. Error propagation in biomass estimation in tropical forests. Methods Ecol Evol 2013;4(2):175–183.

[21] Sileshi GW. A critical review of forest biomass estimation models, common mistakes and corrective measures. For Ecol Manage 2014 10/1;329:237–254.

[22] Temesgen H, Affleck D, Poudel K, Gray A, Sessions J. A review of the challenges and opportunities in estimating above ground forest biomass using tree-level models. Scand J For Res 2015 05/19;30(4):326–335.

[23] Henry M, Picard N, Trotta C, Manlay RJ, Valentini R, Bernoux M, et al Estimating tree biomass of sub-Saharan African forests: A review of available allometric equations. Silva Fenn 2011;45(3):477–569.

[24] Chave J, Réjou-Méchain M, Búrquez A, Chidumayo E, Colgan MS, Delitti WBC, et al Improved allometric models to estimate the aboveground biomass of tropical trees. Global Change Biol 2014;20(10):3177–3190. 10.1111/gcb.12629 24817483

[25] Zanne AE, Lopez-Gonzalez G, Coomes DA, Ilic J, Jansen S, Lewis SL, et al. Global Wood Density Database. 2009.

[26] Ketterings QM, Coe R, Van Noordwijk M, Ambagau' Y, Palm CA. Reducing uncertainty in the use of allometric biomass equations for predicting above-ground tree biomass in mixed secondary forests. For Ecol Manage 2001;146(1–3):199–209.

[27] Feldpausch TR, Lloyd J, Lewis SL, Brienen RJW, Gloor M, Monteagudo Mendoza A, et al Tree height integrated into pantropical forest biomass estimates. Biogeosciences 2012;9(8):3381–3403.

[28] Rutishauser E, Noor'an F, Laumonier Y, Halperin J, Rufi'ie, Hergoualch K, et al Generic allometric models including height best estimate forest biomass and carbon stocks in Indonesia. For Ecol Manage 2013;307:219–225.

[29] Molto Q, H\'erault B, Boreux J-, Daullet M, Rousteau A, Rossi V. Predicting tree heights for biomass estimates in tropical forests–a test from French Guiana. Biogeosciences 2014;11(12):3121–3130.

[30] Sampaio E, Gasson P, Baracat A, Cutler D, Pareyn F, Lima KC. Tree biomass estimation in regenerating areas of tropical dry vegetation in northeast Brazil. For Ecol Manage 2010;259(6):1135–1140.

[31] Kuyah S, Rosenstock TS. Optimal measurement strategies for aboveground tree biomass in agricultural landscapes. Agrofor Syst 2015;8(1):125–133.

[32] Lappi J, Mehtätalo L, Korhonen K. Generalizing sample tree information In: Maltamo M, Maltamo, editors. Forest inventory—methodology & applications: Springer; 2006.

[33] Mehtätalo L. Predicting stand characteristics using limited measurements; 2004.

[34] Greenhill G. Determination of the greatest height consistent with stability that a vertical pole or mast can be made, and of the greatest height to which a tree of given proportions can grow. Proceedings of Cambridge Philosophical Society 1881;4(2).

[35] Meyer HA. A mathematical expression for height curves. J For 1940;38(5):415–420.

[36] Curtis RO. Height-diameter and height-diameter-age equations for second-growth Douglas-fir. For Sci 1967;13(4):365–375.

[37] Huang S, Titus SJ, Wiens DP. Comparison of nonlinear heightâ€“diameter functions for major Alberta tree species. Can J For Res 1992 09/01; 2015/11;22(9):1297–1304.

[38] Zianis D, Muukkonen P, Mäkipää R, Mencuccini M. Biomass and stem volume equations for tree species in Europe. Silva Fennica Monographs 2005;4:1–63.

[39] Mehtätalo L, de-Miguel S, Gregoire TG. Modeling height-diameter curves for prediction. Canadian Journal of Forest Research 2015;45(7):826–837.

[40] Larsen DR, Hann DW. Height-diameter equations for seventeen tree species in southwest Oregon. 1987.

[41] King DA. Allometry and life history of tropical trees. J Trop Ecol 1996;12(1):25–43.

[42] Temesgen H, Gadow Kv. Generalized height-dimater models—An application for major tree species in complex stands of interior British Columbia. European Journal of Forest Research 2004;123(1):45–51.

[43] Eerikäinen K. A multivariate linear mixed-effects model for the generalization of sample tree heights and crown ratios in the Finnish National Forest Inventory. For Sci 2009;55(6):480–493.

[44] Guendehou GHS, Lehtonen A, Moudachirou M, Mäkipää R, Sinsin B. Stem biomass and volume models of selected tropical tree species in West Africa. South For 2012;74(2):77–88.

[45] Jayaraman K, Lappi J. Estimation of height-diameter curves through multilevel models with special reference to even-aged teak stands. For Ecol Manage 2001;142(1–3):155–162.

[46] Calama R, Montero G. Interregional nonlinear height-diameter model with random coefficients for stone pine in Spain. Canadian Journal of Forest Research 2004;34(1):150–163.

[47] Feldpausch TR, Banin L, Phillips OL, Baker TR, Lewis SL, Quesada CA, et al Height-diameter allometry of tropical forest trees. Biogeosciences 2011;8(5):1081–1106.

[48] Zeide B, Vanderschaaf C. The effect of density on the height-diameter relationship. Proceedings of the 11th Biennial Southern Silvicultural Research Conference 2002:463–466.

[49] Sharma M, Parton J. Height-diameter equations for boreal tree species in Ontario using a mixed-effects modeling approach. For Ecol Manage 2007;249(3):187–198.

[50] Pinheiro JC, Bates DM. Mixed-Effects Models in S and S-Plus 2000.

[51] Lappi J, Bailey RL. A height prediction model with random stand and tree parameters: an alternative to traditional site index methods. For Sci 1988;34(4):907–927.

[52] Vargas-Larreta B, Castedo-Dorado F, Álvarez-González JG, Barrio-Anta M, Cruz-Cobos F. A generalized height-diameter model with random coefficients for uneven-aged stands in El Salto, Durango [Mexico]. Forestry 2009;82(4):445–462.

[53] Meng SX, Huang S, Lieffers VJ, Nunifu T, Yang Y. Wind speed and crown class influence the height-diameter relationship of lodgepole pine: Nonlinear mixed effects modeling. For Ecol Manage 2008;256(4):570–577.

[54] Schmidt M, Kiviste A, von Gadow K. A spatially explicit height-diameter model for Scots pine in Estonia. European Journal of Forest Research 2011;130[2]:303–315.

[55] Castedo Dorado F, Diéguez-Aranda U, Barrio Anta M, Sánchez Rodríguez M, von Gadow K. A generalized height-diameter model including random components for radiata pine plantations in northwestern Spain. For Ecol Manage 2006;229(1–3):202–213.

[56] Coble DW, Lee Y-. A mixed-effects height-diameter model for individual loblolly and slash pine trees in East Texas. South J Appl For 2011;35(1):12–17.

[57] Olson DM, Dinerstein E, Wikramanayake ED, Burgess ND, Powell GVN, Underwood EC, et al Terrestrial Ecoregions of the World: A New Map of Life on Earth: A new global map of terrestrial ecoregions provides an innovative tool for conserving biodiversity. Bioscience 2001 11 01;51(11):933–938.

[58] Hijmans RJ, Cameron SE, Parra JL, Jones PG, Jarvis A. Very high resolution interpolated climate surfaces for global land areas. Int J Climatol 2005;25(15):1965–1978.

[59] Peel MC, Finlayson BL, McMahon TA. Updated world map of the Köppen-Geiger climate classification. Hydrology and Earth System Sciences 2007;11(5):1633–1644.

[60] Jones A, Breuning-Madsen H, Brossard M, Dampha A, Deckers J, Dewitte O, et al. Soil Atlas of Africa 2013.

[61] United Nations Development Programme. Additional documents to the UNDP project document PIMS 3970: subprogram for the Centre-West Region. 2010.

[62] Vågen T, Winowiecki L, Desta LT, Tondoh JE. The land degradation surveillance framework field guide 2010.

[63] Boffa JM. Agroforestry Parklands in Sub-Saharan Africa 1999.

[64] Coulibaly-Lingani P, Savadogo P, Tigabu M, Oden P-. Factors influencing people's participation in the forest management program in Burkina Faso, West Africa. Forest Policy and Economics 2011;13(4):292–302.

[65] Sawadogo L, Nygård R, Pallo F. Effects of livestock and prescribed fire on coppice growth after selective cutting of Sudanian savannah in Burkina Faso. Ann For Sci 2002;59(2):185–195.

[66] R Development Core Team. R: A Language and Environment for Statistical Computing 2014.

[67] Pinheiro J, Bates D, DebRoy S, Sarkar D. nlme: Linear and Nonlinear Mixed Effects Models. R package version 3.1–117 2014.

[68] Mehtätalo L. lmfor: Functions for Forest Biometrics 2012.

[69] Zeileis A, Hothorn T. Diagnostic Checking in Regression Relationships. R News 2002;2(3):7–10.

[70] Nygård R, Elfving B. Stem basic density and bark proportion of 45 woody species in young savanna coppice forests in Burkina Faso. Ann For Sci 2000;57(2):143–153.

[71] ICRAF World Agroforestry Centre–CGIAR. Tree Functional Attributes and Ecological Database. http://db.worldagroforestry.org 2015.

[72] Flores O, Coomes DA. Estimating the wood density of species for carbon stock assessments. Methods in Ecology and Evolution 2011;2(2):214–220.

[73] Lappi J. Calibration of height and volume equations with random parameters. For Sci 1991;37(3):781–801.

[74] Banin L, Feldpausch TR, Phillips OL, Baker TR, Lloyd J, Affum-Baffoe K, et al What controls tropical forest architecture? Testing environmental, structural and floristic drivers. Global Ecol Biogeogr 2012;21(12):1179–1190.

[75] Baker TR, Phillips OL, Laurance WF, Pitman NCA, Almeida S, Arroyo L, et al Do species traits determine patterns of wood production in Amazonian forests? Biogeosciences 2009;6(2):297–307.

